# A Low Serum Bicarbonate Concentration as a Risk Factor for Mortality in Peritoneal Dialysis Patients

**DOI:** 10.1371/journal.pone.0082912

**Published:** 2013-12-12

**Authors:** Tae Ik Chang, Hyung Jung Oh, Ea Wha Kang, Tae-Hyun Yoo, Sug Kyun Shin, Shin-Wook Kang, Kyu Hun Choi, Dae Suk Han, Seung Hyeok Han

**Affiliations:** 1 Department of Internal Medicine, NHIS Medical Center, Ilsan Hospital, Goyangshi, Gyeonggi–do, Republic of Korea; 2 Department of Internal Medicine, College of Medicine, Yonsei University, Seoul, Republic of Korea; 3 Brain Korea 21 for Medical Science, Severance Biomedical Science Institute, Yonsei University, Seoul, Republic of Korea; University of Florida, United States of America

## Abstract

**Background and Aim:**

Metabolic acidosis is common in patients with chronic kidney disease and is associated with increased mortality in hemodialysis patients. However, this relationship has not yet been determined in peritoneal dialysis (PD) patients.

**Methods:**

This prospective observational study included a total of 441 incident patients who started PD between January 2000 and December 2005. Using time-averaged serum bicarbonate (TA-Bic) levels, we aimed to investigate whether a low serum bicarbonate concentration can predict mortality in these patients.

**Results:**

Among the baseline parameters, serum bicarbonate level was positively associated with hemoglobin level and residual glomerular filtration rate (GFR), while it was negatively associated with albumin, C-reactive protein (CRP) levels, peritoneal Kt/V urea, and normalized protein catabolic rate (nPCR) in a multivariable linear regression analysis. During a median follow-up of 34.8 months, 149 deaths were recorded. After adjustment for age, diabetes, coronary artery disease, serum albumin, ferritin, CRP, residual GFR, peritoneal Kt/V urea, nPCR, and percentage of lean body mass, TA-Bic level was associated with a significantly decreased risk of mortality (HR per 1 mEq/L increase, 0.83; 95% CI, 0.76-0.91; *p* < 0.001). In addition, compared to patients with a TA-Bic level of 24-26 mEq/L, those with a TA-Bic level < 22 and between 22-24 mEq/L conferred a 13.10- and 2.13-fold increased risk of death, respectively.

**Conclusions:**

This study showed that a low serum bicarbonate concentration is an independent risk factor for mortality in PD patients. This relationship between low bicarbonate levels and adverse outcome could be related to enhanced inflammation and a more rapid loss of RRF associated with metabolic acidosis. Large randomized clinical trials to correct acidosis are warranted to confirm our findings.

## Introduction

A low serum bicarbonate concentration, manifested as an important clinical disturbance of metabolic acidosis, is common in end-stage renal disease (ESRD) and is believed to be an important cause of many deleterious metabolic consequences including protein-energy wasting, inflammation, bone disease, and disturbance in endocrine function [1–5]. The unfavorable effects of metabolic acidosis can explain the increased mortality in patients undergoing maintenance hemodialysis (HD), but the underlying mechanisms are still in need of clarification. In addition, the optimal bicarbonate level to avoid adverse clinical outcomes is largely unknown [6–9].

 Along with HD, peritoneal dialysis (PD) is an established treatment modality in ESRD and approximately 150,000 patients worldwide are being maintained on PD [10]. Given the continuous provision of dialysis treatment with PD, it can be presumed that PD may be more effective in correcting metabolic acidosis than HD; thus, the effect of metabolic acidosis on clinical outcomes may differ between the two dialysis modalities. However, few studies have examined the relationship between serum bicarbonate level and risk of death in PD patients. Therefore, the intent of this study was to investigate whether low serum bicarbonate levels can predict mortality in a large prospective cohort of incident patients undergoing PD.

## Methods

### Ethics statement

The study was carried out in accordance with the Declaration of Helsinki and approved by the Institutional Review Board of Ilsan Hospital Clinical Trial Center. We obtained informed written consent from all participants involved in our study.

### Patients

**Figure 1:**
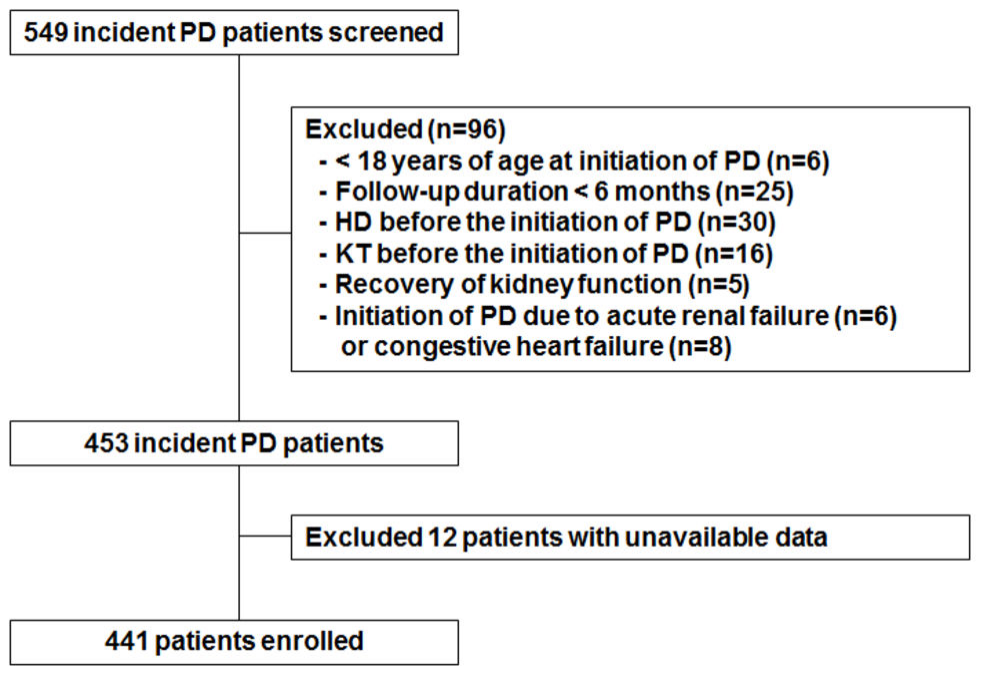
Flow chart of participants in the cohort. PD, peritoneal diaysis; HD, hemodialysis; KT, kidney transplant.

The study population included 549 ESRD patients who started PD at Yonsei University Severance Hospital or NHIS Ilsan hospital between January 2000 and December 2005. All patients underwent urea kinetic studies including residual renal function (RRF) within three months of PD initiation. We excluded patients < 18 years of age at initiation of PD, patients that had less than 6 months of follow-up, and patients that had been on HD or received a kidney transplant before the initiation of PD. Patients that recovered kidney function or started PD for other reasons, such as acute renal failure or congestive heart failure, were also excluded from the analysis. Therefore, this prospective observational study included a total of 441 incident patients (Figure 1).

### Data collection

Demographic and clinical data were collected at the beginning of PD. These included age, gender, body mass index (BMI) calculated as weight/(height)^2^, cause of ESRD, prevalence of diabetes and coronary artery disease (CAD). Laboratory data obtained at the time of dialysis adequacy measurement were considered baseline values and included serum bicarbonate concentrations, blood urea nitrogen, serum creatinine, total cholesterol, serum albumin, serum C-reactive protein (CRP) levels, Kt/V urea, percentage of lean body mass (%LBM), normalized protein catabolic rate (nPCR), and residual glomerular filtration rate (GFR). Residual GFR was calculated as the average urea and creatinine clearance from a 24-h urine collection [11]. Serum total CO_2_, which is generally used as an indirect measure of serum bicarbonate concentration [2], was measured by an electrode-based method (UniCel DXC 800; Beckman Coulter, Inc., CA, USA) and recorded longitudinally throughout the follow-up period. Time-averaged serum bicarbonate (TA-Bic) was calculated as an average of the mean of bicarbonate measurements every 3-month period.

### Study outcomes

The study participants were followed until December 31, 2011. The primary outcome parameter was all-cause and cardiovascular mortality.

### Statistical analysis

All values are expressed as the mean ± standard deviation or percentages. Statistical analyses were performed using SPSS for Windows version 13.0 (SPSS, Inc., Chicago, IL, USA). Data were analyzed using Student’s *t*–test and the Chi-square test, and ANOVA was used for multiple comparisons. The Kolmogorov-Smirnov test was used to determine the normality of the distribution of parameters. If data did not show a normal distribution, they were expressed as the median and interquartile range (or after log-transformation) and were compared using the Mann–Whitney test or Kruskal–Wallis test. The relationships between serum bicarbonate and continuous variables were examined by Pearson’s correlation coefficient, and categorical variables were examined using Spearman’s *R* test. Multiple linear regression analysis was performed to identify the determinants of serum bicarbonate levels. Survival rate was compared among 3 groups based on TA-Bic levels (< 22, 22 to 24, and ≥ 24 mEq/L) using the Kaplan-Meier method and the log-rank test. Data for switch to HD, kidney transplantation, and loss to follow-up were censored in the analysis. Patients who died within the first 3 months after converting to HD or receiving a kidney graft were considered deaths related to PD. To determine risk factors for mortality, multivariate Cox regression was performed, and all significant covariates from the univariate analysis were included. The results are expressed as a hazard ratio (HR) and 95% confidence interval (CI). A *p*–value less than 0.05 was considered statistically significant.

## Results

### Patient characteristics

The mean age of the patients was 59.2 years (range, 22 to 85 years), 54.4% were males, and patients were on PD for a mean duration of 43.2 months (range, 6 to 142 months). The prevalence of diabetes and CAD was 51.5% and 15.9%, respectively. The mean TA-Bic level was 25.9 ± 2.4 mEq/L (median, 25.8 mEq/L; range, 16.6 to 33.9 mEq/L). Table 1 details the baseline characteristics of the 441 patients categorized into 5 groups by TA-Bic level: < 22 mEq/L, ≥ 28 mEq/L, and every 2.0 mEq/L increment of bicarbonate in between. Serum hemoglobin (*p* for trend < 0.001), total cholesterol concentrations (*p* for trend = 0.024), and residual GFR (*p* for trend = 0.015) were higher at higher TA-Bic level, whereas serum albumin (*p* for trend = 0.001), peritoneal Kt/V urea (*p* for trend < 0.001), and serum CRP levels (*p* for trend < 0.001) were lower.

**Table 1:** Baseline characteristics of the study subjects stratified by time-averaged serum bicarbonate.

	Time-averaged serum bicarbonate (mEq/L; Number of subjects)					
	<22 (n=18)	22 to <24 (n=83)	24 to <26 (n=127)	26 to <28 (n=128)	≥28 (n=85)	*p* for trend
Age (years)	64.1 ± 11.5	57.4 ± 14.0	57.2 ± 14.0	60.7 ± 13.7	60.8 ± 13.5	0.182
Gender (Male)	11 (61.1)	41 (49.4)	69 (54.3)	69 (53.9)	50 (58.8)	0.463
Body mass index (kg/m^2^)	21.6 ± 2.6	22.7 ± 3.3	23.6 ± 11.5	22.6 ± 2.9	22.5 ± 3.3	0.820
Presence of diabetes mellitus	8 (44.4)	38 (45.8)	62 (48.8)	71 (55.5)	48 (56.5)	0.076
Presence of coronary artery disease	3 (16.7)	10 (12.0)	14 (11.0)	28 (21.9)	15 (17.6)	0.113
Laboratory findings						
Hemoglobin (g/dL)	10.4 ± 1.5	10.3 ± 1.6	10.8 ± 1.5	11.0 ± 1.5	11.4 ± 1.3	<0.001
Serum albumin (g/dL)	3.2 ± 0.6	3.2 ± 0.5	3.3 ± 0.5	3.1 ± 0.5	3.0 ± 0.6	<0.001
Total cholesterol (mg/dL)	170.0 [105-250]	177.0 [6-383]	183.0 [94-273]	181.0 [66-451]	178.0 [95-334]	0.024
Serum ferritin (ng/mL)	286 [55-1429]	270.2 [15-1693]	174.3 [7-1711]	124.7 [14-1680]	157.6 [21-1373]	0.062
CRP (mg/dL)	0.3 [0.01-8.1]	0.2 [0.01-36.2]	0.1 [0.01-36.3]	0.1 [0.01-9.1]	0.02 [0.01-4.7]	<0.001
Residual GFR (mL/min/1.73m^2^)	3.1 [0.7-11.2]	5.1 [0.5-15.8]	5.4 [0.6-18.8]	5.5 [0.6-26.9]	5.9 [0.6-36.9]	0.015
Total Kt/Vurea	2.3 ± 0.6	2.7 ± 0.9	2.7 ± 0.8	2.6 ± 0.9	2.4 ± 0.6	0.106
Peritoneal Kt/V urea	1.6 ± 0.6	1.6 ± 0.5	1.6 ± 0.6	1.4 ± 0.5	1.2 ± 0.5	<0.001
nPCR (g/Kg/day)	0.8 ± 0.2	1.0 ± 0.2	1.0 ± 0.3	1.0 ± 0.3	1.0 ± 0.2	0.533
Lean body mass (% body weight)	55.3 ± 13.4	66.6 ± 14.2	65.8 ± 13.6	63.1 ± 12.7	63.5 ± 14.9	0.931

Values for categorical variables are given as number (percentage); values for continuous variables are given as mean ± standard deviation or median [interquartile range]. CRP, C-reactive protein; GFR, glomerular filtration rate; nPCR, normalized protein catabolic rate.

### Factors associated with baseline serum bicarbonate

Correlation analyses were performed to identify factors associated with baseline serum bicarbonate level (Table 2). There was no correlation between age, BMI, presence of preexisting CAD, serum ferritin, total Kt/V urea, %LBM, and serum bicarbonate. In contrast, baseline serum bicarbonate level positively correlated with male gender (ρ = 0.127; *p* = 0.007), presence of diabetes (ρ = 0.125; *p* = 0.009), hemoglobin (ρ = 0.226; *p* < 0.001), total cholesterol levels (ρ = 0.130; *p* = 0.006), and residual GFR (ρ = 0.158; *p* = 0.001), whereas it inversely correlated with serum albumin (ρ = -0.227; *p* < 0.001), serum CRP levels (ρ = -0.182; *p* < 0.001), peritoneal Kt/V urea (ρ = -0.267; *p* < 0.001), and nPCR (ρ = -0.177; *p* < 0.001). Multivariate linear regression analysis adjusted for these factors revealed that serum hemoglobin (β = 0.153; *p* = 0.001), serum albumin (β = -0.275; *p* < 0.001), CRP levels (β = -0.148; *p* = 0.002), residual GFR (β = 0.118; *p* = 0.027), peritoneal Kt/V urea (β = -0.120; *p* = 0.028), and nPCR (β = -0.121; *p* = 0.018) were independently associated with baseline serum bicarbonate (Table 2).

**Table 2:** Univariate and multivariate associations between baseline serum bicarbonate level and patient characteristics.

	Univariate				Multivariate		
	*ρ*	*p*				β	*p*	
Age (years)	0.049	0.303				－	－	
Gender (Male)	0.127	0.007				0.040	0.413	
Body mass index (kg/m^2^)	-0.020	0.677				—	－	
Presence of diabetes	0.125	0.009				0.015	0.753	
Presence of coronary artery disease	0.086	0.071				—	－	
Hemoglobin (g/dL)	0.226	<0.001				0.153	0.001	
Serum albumin (g/dL)	-0.227	<0.001				-0.275	<0.001	
Total cholesterol (mg/dL)^a^	0.130	0.006				0.079	0.098	
Ferritin (ng/mL)^a^	-0.082	0.086				—	—	
CRP (mg/dL)^a^	-0.182	<0.001				-0.148	0.002	
Residual GFR (mL/min/1.73m^2^)^a^	0.158	0.001				0.118	0.027	
Total Kt/Vurea	-0.069	0.151						
Peritoneal Kt/V urea	-0.267	<0.001				-0.120	0.028	
nPCR (g/Kg/day)	-0.177	<0.001				-0.121	0.018	
Lean body mass (% body weight)	-0.028	0.868				—	－	

CRP, C-reactive protein; GFR, glomerular filtration rate; nPCR, normalized protein catabolic rate. **^*a*^**Data for total cholesterol, ferritin, CRP, and residual GFR were log transformed.

### Metabolic acidosis as a predictor of mortality

During follow-up, 149 deaths were recorded, and the median survival period was 34.8 months (range, 6.0 to 142.2 months). Infection (37.6%) was the most common cause of death in this study, followed by cardiovascular disease (36.2%).

**Figure 2:**
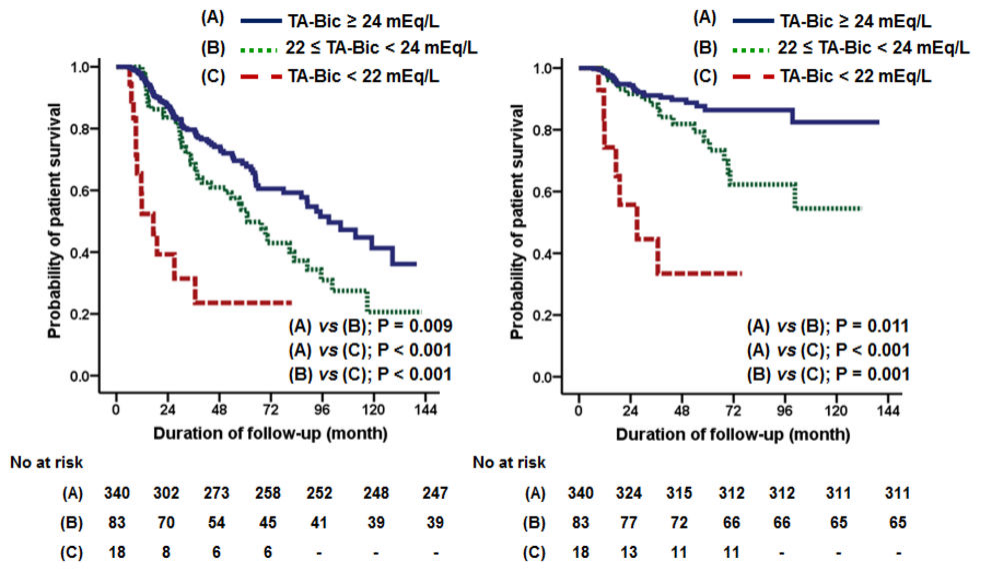
Kaplan-Meier plots for all-cause (right) and cardiovascular (left) mortality based on the level of time-averaged serum bicarbonate (TA-Bic).

 In the unadjusted Cox proportional hazards models, TA-Bic level significantly predicted all-cause mortality (HR per 1 mEq/L increase, 0.88; 95% CI, 0.81-0.95; *p* = 0.001). A Kaplan-Meier plot also showed that time to death was significantly longer in patients with a higher TA-Bic level compared to patients with a TA-Bic level < 22 mEq/L (Figure 2). In addition, age, diabetes, preexisting CAD, serum albumin, ferritin, CRP, residual GFR, peritoneal Kt/V urea, nPCR, and %LBM were revealed as predictors of mortality. In the multivariable analysis after adjustment of these factors, increased TA-Bic level was associated with a significantly decreased risk of death (HR per 1 mEq/L increase, 0.83; 95% CI, 0.76-0.91; *p* < 0.001) (Table 3). A similar association was observed between TA-Bic level and cardiovascular mortality (Figure 2 and Table 3); however, there was no significant relationship between TA-Bic level and infection-related death (data not shown).

**Table 3:** Cox regression analysis for all-cause and cardiovascular mortality.

	All-cause mortality								Cardiovascular mortality						
	Univariate				Multivariate				Univariate				Multivariate		
	HR	(95% CI)	*p*		HR	(95% CI)	*p*		HR	(95% CI)	*p*		HR	(95% CI)	*p*
Age (years)	1.08	(1.06-1.10)	<0.001		1.07	(1.04-1.09)	<0.001		1.05	(1.03-1.08)	<0.001		1.04	(1.01-1.07)	0.004
Gender (Male)	1.25	(0.90-1.73)	0.183		—	—	—		1.48	(0.85-2.56)	0.164		—	—	—
Body mass index (kg/m^2^)	1.00	(0.98-1.02)	0.971		—	—	—		0.98	(0.92-1.05)	0.627		—	—	—
Presence of diabetes	1.82	(1.31-2.54)	<0.001		1.59	(1.07-2.34)	0.022		1.83	(1.05-3.17)	0.032		1.28	(0.69-2.37)	0.433
Presence of coronary artery disease	2.29	(1.55-3.39)	<0.001		1.24	(0.80-1.93)	0.332		6.35	(3.63-11.13)	<0.001		4.08	(2.23-7.46)	<0.001
Hemoglobin (g/dL)	0.92	(0.82-1.02)	0.115		—	—	—		0.91	(0.77-1.09)	0.310		—	—	—
Serum albumin (g/dL)	0.28	(0.21-0.38)	<0.001		0.36	(0.25-0.52)	<0.001		0.34	(0.21-0.57)	<0.001		0.36	(0.20-0.64)	<0.001
Total cholesterol (mg/dL)^a^	0.85	(0.26-2.82)	0.848		—	—	—		0.44	(0.05-4.26)	0.481		—	—	—
Serum ferritin (ng/mL)^a^	2.94	(2.03-4.26)	<0.001		1.25	(0.85-1.83)	0.264		2.60	(1.40-4.81)	0.002		1.39	(0.73-2.63)	0.318
CRP (mg/dL)^a^	1.75	(1.39-2.19)	<0.001		1.15	(0.90-1.47)	0.262		1.43	(0.99-2.08)	0.058		—	—	—
TA-Bic (mEq/L)	0.88	(0.81-0.95)	0.001		0.83	(0.76-0.91)	<0.001		0.81	(0.71-0.92)	0.001		0.78	(0.69-0.88)	<0.001
Residual GFR (mL/min/1.73m^2^)^a^	0.28	(0.18-0.43)	<0.001		0.43	(0.22-0.84)	0.013		0.25	(0.13-0.52)	<0.001		0.49	(0.22-1.09)	0.077
Peritoneal Kt/Vurea	1.40	(1.05-1.86)	0.021		0.85	(0.57-1.28)	0.850		1.28	(0.80-2.04)	0.303		—	—	—
nPCR (g/Kg/day)	0.18	(0.09-0.38)	<0.001		0.62	(0.38-1.79)	0.820		0.22	(0.07-0.72)	0.012		0.87	(0.28-2.66)	0.804
Lean body mass (% body weight)	0.96	(0.94-0.97)	<0.001		1.00	(0.98-1.02)	0.969		0.97	(0.95-0.99)	0.001		1.01	(0.98-1.04)	0.391

CRP, C-reactive protein; TA-Bic, time-averaged serum bicarbonate; GFR, glomerular filtration rate; nPCR, normalized protein catabolic rate, HR, hazard ratio; CI, confidence interval. **^*a*^**Data for total cholesterol, ferritin, CRP, and residual GFR were log transformed.

**Figure 3:**
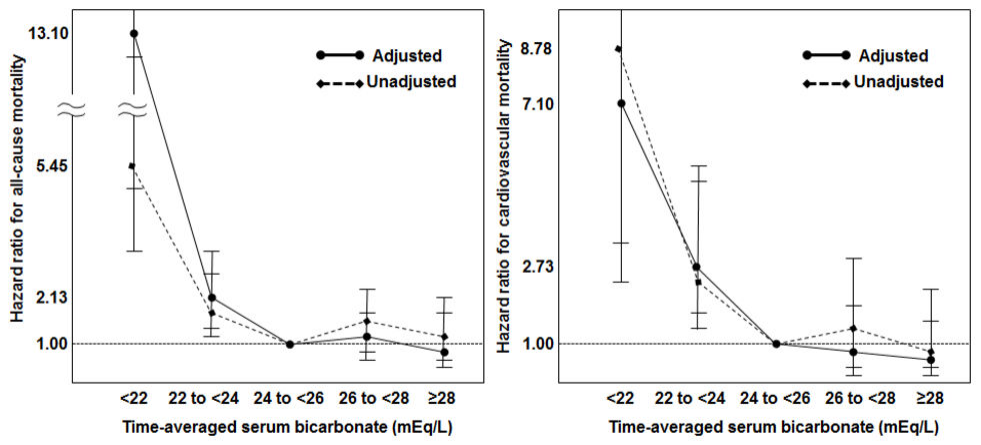
Hazard ratios for all-cause (right) and cardiovascular (left) mortality in each of the five time-averaged serum bicarbonate (TA-Bic) subgroups. Error bars indicate 95% confidence intervals for unadjusted and adjusted models. The subgroup with TA-Bic levels of 24-26 mEq/L was the reference group in all models.

 We also conducted a separate multivariate analysis in which TA-Bic levels were categorized into 5 groups as presented in Table 1. Compared to patients with a TA-Bic level of 24-26 mEq/L, patients with a TA-Bic level < 22 mEq/L and 22-24 mEq/L conferred a 13.10- and 2.13-fold increased risk of all-cause mortality, respectively. HRs for cardiovascular death were 7.10 and 2.73 in patients with a TA-Bic level < 22 mEq/L and 22-24 mEq/L versus those with a TA-Bic level of 24-26 mEq/L. However, no further survival advantage was observed with TA-Bic level greater than 26 mEq/L (Figure 3).

## Discussion

In this study, we aimed to delineate the relationship between serum bicarbonate levels and mortality in our ESRD cohort of patients with PD. We showed that serum bicarbonate level exhibited a significant inverse association with serum albumin and CRP levels, while it positively correlated with RRF. In addition, low TA-Bic level independently predicted all-cause and cardiovascular mortality in these patients, suggesting that metabolic acidosis exerts a detrimental effect; thus, patients with a low bicarbonate level should be considered for proper treatment even though they are receiving dialysis therapy.

 Unlike conventional HD, PD provides continuous dialysis, which leads to the assumption that metabolic acidosis is uncommon in patients with PD. To date, the prevalence of metabolic acidosis in PD patients has not been fully explored, and several studies have reported that the proportion of patients with serum bicarbonate < 22 mEq/L varies widely from 10 to 25% [6,12–14]. Of note, most previous studies conducted cross-sectional surveys using a single measurement of serum bicarbonate concentration [12–14]. Given that such a value measured at a single specific time-point does not represent acid-base state during the entire period of dialysis, TA-Bic is a more reliable parameter to reflect acidosis. In fact, in this study, there were only 4.1% of patients with a TA-Bic level < 22 mEq/L, while 14.5% of patients had a serum bicarbonate level < 22 mEq/L at the time of PD initiation. Surprisingly, a recent large cohort study using the DaVita database reported that 25% of patients had a TA-Bic level < 22 mEq/L, which was unexpectedly high [6]. The authors postulated that the delayed measurements performed in samples shipped overnight to a central laboratory might be responsible for the higher prevalence of low serum bicarbonate level in their study. It should be noted that artificial reduction in the total CO_2_ concentration might result from increased lactic acid production due to delays in the centrifugation of blood and in analysis [15]. Thus, the presence of metabolic acidosis should be verified by on-site measurements. To minimize biased results, the two centers in the present study used the same analyzer to measure biochemical parameters including bicarbonate level, and all samples were analyzed within 4 hours after blood sampling.

 Our findings provide concrete evidence that low serum bicarbonate level is associated with adverse outcome in PD patients as well as in HD patients and chronic kidney disease patients prior to dialysis therapy [6–9,16]. The robust analyses of our study clearly show that low serum bicarbonate level is an independent predictor of mortality in the multivariate Cox proportional hazards model adjusted for potentially multiple confounding covariates such as age, comorbidities, serum albumin, ferritin and CRP levels, RRF, Kt/V urea, nPCR, and %LBM. In addition, patients with a TA-Bic levels < 22 mEq/L and 22 to < 24 mEq/L had adjusted hazards ratios for all-cause mortality of 13.10 and 2.13, respectively, compared to reference patients with a TA-Bic level between 24 and 26 mEq/L. Relevant to our finding is a recent observation by Vashistha et al. [6], showing that TA-Bic levels < 19 or 21 to < 22 mEq/L were associated with an 18% and 13% higher risk of death in PD patients, respectively (reference group: TA-Bic 24 to < 25 mEq/L).

However, there is no consensus about the optimal bicarbonate level in ESRD patients, and it is also uncertain whether target serum bicarbonate level should be individualized by dialysis modality. In an analysis of 7,140 HD patients based on data from the Dialysis Outcomes and Practice Pattern Study, the lowest risk for mortality was associated with midweek pre-dialysis bicarbonate levels between 20 and 21 mEq/L, while an increased risk was observed in patients with high (> 27 mEq/L) or low (< 17 mEq/L) bicarbonate levels [7]. Another study of 56,385 HD patients using the DaVita database showed a reverse J-shaped pattern between serum bicarbonate and mortality, where serum bicarbonate levels > 22 mEq/L were associated with lower mortality risk. However, bicarbonate levels below this measure showed a consistently increased risk [8]. Using the same database, a recent study by Vashistha et al. [6] recruited the largest cohort to date and obtained a similar result. Taken together, among patients treated with HD, a low pre-dialysis serum bicarbonate level, particularly < 22 mEq/L, appears to correlate with poor survival. In keeping with these findings, clinical practice guidelines from the National Kidney Foundation Disease Outcomes Quality Initiative recommend a serum bicarbonate level ≥ 22 mEq/L, irrespective of dialysis modality [17,18].

Nevertheless, there is a lack of compelling evidence about whether serum bicarbonate level should be maintained at > 22 mEq/L in patients with PD. Only two studies including ours have addressed this issue in the PD population and have shown conflicting results. In the aforementioned study by Vashistha et al. [6], they extended the cohort to PD patients and found that TA-Bic levels with 22 to < 24 mEq/L exhibited a comparable risk of mortality compared to TA-Bic levels ≥ 24 mEq/L. In contrast, we showed that TA-Bic levels with 22 to < 24 mEq/L were associated with a significantly higher mortality risk than TA-Bic levels ≥ 24 mEq/L (Figure 2 and 3). The observational nature of our study does not fully explain this discrepancy, but it is possible that a relatively low proportion of patients with TA-Bic levels < 24 mEq/L led to the biased results. However, it should be noted that, as previously mentioned, delayed sample analysis may have falsely decreased bicarbonate levels in the study by Vashistha et al. [6]; thus, their findings should be interpreted with caution.

 Interestingly, the United Kingdom Renal Association recommends serum bicarbonate levels to be within the normal range for PD patients but provides greater latitude for HD patients (target pre-dialysis serum bicarbonate levels 18–24 mEq/L) [19,20]. The guidelines suggest a higher threshold of bicarbonate in PD patients than in HD patients. A rationale for this can be provided by two randomized controlled trials indicating that oral bicarbonate supplement or dialysis fluid with a 40 mmol buffer capacity significantly improved nutritional status compared to a placebo group, although the mean bicarbonate levels in patients treated with placebo were 24.7 and 23.0 mEq/L in the two studies, respectively [21,22]. Our finding supports the target serum bicarbonate levels in PD patients recommended by the recent UK guidelines.

The underlying mechanisms for increased mortality in PD patients with lower serum bicarbonate are unclear. The possible mechanisms include chronic inflammation [23–25] and loss of RRF [26–28] which have been established to be a powerful predictor of mortality in patients on dialysis. In our study, serum bicarbonate level was inversely associated with serum CRP levels, whereas it positively correlated with RRF, therefore it is postulated that acidosis *per se* could be worsened by inflammation or a more rapid loss of RRF. On the other hand, we found that serum bicarbonate level negatively correlated with serum albumin concentration as well as nPCR, but was not related to other nutritional parameters such as BMI, serum total cholesterol concentration, or %LBM. These inconsistent results on relationship between bicarbonate level and various nutritional parameters make it difficult to conclude that such a negative correlation between serum bicarbonate level and serum albumin found in our study is a reflection of increased protein intake, which will also lead to a better nutritional status. There are, however, controversial opinions about the effect of serum bicarbonate on nutrition. Good dietary intake is usually accompanied by high protein intake, which increases acid load and can be a cause of acidosis in uremic patients [3,7,14,23]. Conversely, metabolic acidosis *per se* can have a negative impact on nutritional status including increased proteolysis, decreased protein synthesis, endocrine abnormalities including insulin resistance, and inflammation [1–4,29–33]. More randomized, controlled trials are required to explore if interventions to correct metabolic acidosis can improve hard clinical outcomes such as mortality, and to clarify if such an improvement is mediated through the attenuation of inflammation and malnutrition.

There are several limitations in the present study. First, this is an observational study with a relatively small sample size. Hence, causality of our findings needs further confirmation. Second, it is possible that more sick patients were included in the group of TA-Bic < 22 mEq/L. We tried to minimize risk of inclusion of such more seriously ill patients at the initiation of PD by extending follow-up period up to 6 months. In fact, most epidemiologic studies follow 90-day rule for inclusion of incident-based patients, meaning that patients must have survived the first 90 days on dialysis to be eligible for analysis [34]. Nevertheless, we could not entirely exclude possibility that patients with TA-Bic < 22 mEq/L might be in more seriously ill condition. Third, other data representing overall nutritional status such as subjective global assessment, anthropometry, and dietary protein intake were not available for the analysis. Importantly, a low serum bicarbonate level in this study may not be diagnostic of metabolic acidosis, because it represents the state of low arterial pH (acidemia) and reduced serum bicarbonate concentration, accompanied by decreased P_C_O_2_. Unfortunately, data of arterial gas analyses such as arterial pH and P_C_O_2_ were unavailable in our study, thus it is uncertain whether a low serum bicarbonate level is associated with combined acidemia or not. However, given the loss of buffering capacity by the kidney in ESRD patients, they are more likely to have metabolic acidosis than respiratory alkalosis, which is another clinical condition that causes decreased bicarbonate level. Despite these limitations, we adjusted various factors associated with nutritional status, inflammation, and RRF and found an adverse effect of a low serum bicarbonate level on clinical outcome.

## Conclusion

This study showed that a low serum bicarbonate level was an independent risk factor for mortality in PD patients. The relationship between low bicarbonate levels and adverse outcome may be related to enhanced inflammation and a more rapid loss of RRF associated with metabolic acidosis. Large randomized clinical trials to correct acidosis are warranted to confirm our findings.
